# A review of second‐order blind identification methods

**DOI:** 10.1002/wics.1550

**Published:** 2021-02-07

**Authors:** Yan Pan, Markus Matilainen, Sara Taskinen, Klaus Nordhausen

**Affiliations:** ^1^ Department of Mathematics and Statistics University of Jyväskylä Finland; ^2^ Turku PET Centre Turku University Hospital and University of Turku Finland; ^3^ Institute of Statistics and Mathematical Methods in Economics, TU Vienna Austria

**Keywords:** blind source separation, dimension reduction, joint diagonalization, multivariate time series

## Abstract

Second‐order source separation (SOS) is a data analysis tool which can be used for revealing hidden structures in multivariate time series data or as a tool for dimension reduction. Such methods are nowadays increasingly important as more and more high‐dimensional multivariate time series data are measured in numerous fields of applied science. Dimension reduction is crucial, as modeling such high‐dimensional data with multivariate time series models is often impractical as the number of parameters describing dependencies between the component time series is usually too high. SOS methods have their roots in the signal processing literature, where they were first used to separate source signals from an observed signal mixture. The SOS model assumes that the observed time series (signals) is a linear mixture of latent time series (sources) with uncorrelated components. The methods make use of the second‐order statistics—hence the name “second‐order source separation.” In this review, we discuss the classical SOS methods and their extensions to more complex settings. An example illustrates how SOS can be performed.

This article is categorized under:

Statistical Models > Time Series Models
Statistical and Graphical Methods of Data Analysis > Dimension Reduction
Data: Types and Structure > Time Series, Stochastic Processes, and Functional Data

## Graphical abstract

Second‐order source separation, which is a variant of blind source separation (BSS), recovers unobservable latent times series from their observed mixtures.

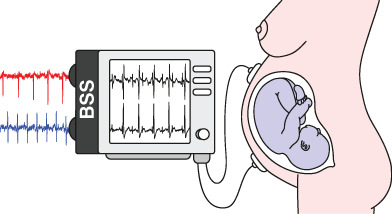

## 1 INTRODUCTION

Many fields of science (e.g., engineering, finance, and biomedicine) collect nowadays multivariate time series data which consist of correlated component series. As the data are often very high‐dimensional, fitting multivariate time series models with large number of parameters is computationally impractical unless the models are noticeably simplified. Blind source separation (BSS) is a widely used multivariate method for extracting latent, uncorrelated source signals from observed multivariate time series data. BSS often provide a deeper inside into the data by revealing hidden structures and the methods can also be used as a preprocessing step to reduce the dimension of the problem at hand. It is, for example, much easier to model the uncorrelated components individually and transform the results back to the original scale than fitting a multivariate model at the original scale.

BSS methods originate from the signal processing literature (see Comon & Jutten, 2010, and references therein), but have gained recently increasing interest also among statisticians (see Nordhausen & Oja, 2018, for example). In the blind source separation model, under consideration in this review, we assume that an observable *p*‐variate time series ***x*** = (***x***_*t*_)_*t* ∈ ℤ_ is a linear instantaneous mixture of a *p*‐variate latent vector ***z*** = (***z***_*t*_)_*t* ∈ ℤ_ with uncorrelated and possibly even independent components. The aim of BSS is then to recover original latent vectors ***z*** (sources) given the observed time series data. In this article, we concentrate on the second‐order separation (SOS) model, a submodel of BSS, where we assume that the components of ***z*** are uncorrelated. Notice that the SOS model does not make any other structural assumptions on the latent vectors (such as a special time series model structure).

The starting point for the development of SOS methods was the algorithm for multiple unknown signals extraction (AMUSE, Tong et al., 1990) and its generalization second‐order blind identification (SOBI, Belouchrani et al., 1997), which make use of the temporal dependence of the components via joint diagonalization of one or several autocovariance matrices, respectively. Some recent applications of SOBI include automatic removal of artifacts in EEG data (Joyce et al., 2004), finding interesting signals in brain imaging data (Tang, 2010; Tang, Sutherland, & McKinney, 2005), separating vibrations caused by underground traffic (Popescu & Manolescu, 2007), and forecasting of wind speed (Firat et al., 2010). AMUSE and SOBI are well‐established and widely studied methods for the separation of uncorrelated weakly stationary time series (Miettinen et al., 2012, 2014, 2016). However, for other BSS models and different time series settings, these methods need to be modified accordingly. The aim of this review is twofold. First, we review the classical AMUSE and SOBI methods and discuss their statistical properties in detail. Second, we give a comprehensive review of recent generalizations of AMUSE and SOBI methods which are developed to perform well for more complex BSS models.

The structure of this overview is as follows. In Section 2, we review the original AMUSE and SOBI methods and discuss the statistical properties of corresponding signal separation matrices. In Section 3, we list some limitations the original SOBI method has and go through several modifications and generalizations of the method. In Section 4, we demonstrate in an application how SOBI fails to separate electrocardiography (ECG) recordings and why extensions of SOBI are relevant. Section 5 lists available algorithms and software for the methods discussed. Finally, in Section 6, we conclude the article with a brief summary and discuss possible future work in this area.

## 2 AMUSE AND SOBI

Let us first recall the original SOBI algorithm and its special case, the algorithm for multiple unknown signals extraction (AMUSE). These two methods were proposed by Belouchrani et al. (1997) and Tong et al. (1990), respectively, to solve the second‐order source separation (SOS) problem. Hence, consider a second‐order separation model

(1) $$x_{t}=\mu +{\mathrm{Az}}_{t},\;t\in \mathbb{Z},$$

where ***μ*** ∈ ℝ^*p*^ is a location vector and ***A*** ∈ ℝ^*p* × *p*^ is the mixing matrix. For the *p*‐variate latent time series ***z*** = (***z***_*t*_)_*t* ∈ ℤ_ we assume that

(2) $$E\;\left(z_{t}\right)=0\;\text{and}\;\mathrm{Cov}\;\left(z_{t}\right)=I_{p},$$

and that the *p* processes in ***z*** ∈ ℝ^*p*^ are assumed to be jointly weakly stationary and mutually uncorrelated meaning that

(3) $$E\;\left(z_{t}z_{t+\tau }^{\prime }\right)=E\;\left(z_{t+\tau }z_{t}^{\prime }\right)=D_{\tau },$$

for all lags *τ* > 0, where ***D***_*τ*_ is a *p* × *p* diagonal matrix. We also assume that all latent sources have different autocovariance structures. Notice that in this article, we assume a real‐valued SOS model. However, many of the presented methods are or can be extended to the case of complex‐valued times series. The aim of SOS is to find a signal separation matrix ***W*** ∈ ℝ^*p* × *p*^ such that the components of ***W*** (***x***_*t*_ − ***μ***) = ***JPz***_*t*_ are standardized and mutually uncorrelated, for a sign‐change matrix J∈J and a permutation matrix P∈P. Here J denotes a set of *p* × *p* sign‐change matrices, whereas P is a set of *p* × *p* permutation matrices.

Both AMUSE and SOBI proceed as follows. Write the standardized time series as $x_{t}^{\mathrm{st}}=\mathrm{Cov}\;{\left(x_{t}\right)}^{-1/2}\left(x_{t}-\mu \right)$, where Cov (***x***_*t*_)^−1/2^ is the symmetric square root of Cov (***x***_*t*_). Then there exists an orthogonal matrix ***U*** ∈ ℝ^*p* × *p*^ such that $x_{t}^{\mathrm{st}}={\mathrm{Uz}}_{t}$ (Cardoso & Souloumiac, 1993; Miettinen et al., 2015). Thus, after standardization, the estimation problem reduces to finding an orthogonal matrix ***U*** only. The signal separation matrix that solves the SOS problem is then given by ***W*** = ***U*** Cov (***x***_*t*_)^−1/2^.

AMUSE (Tong et al., 1990) uses the cross‐autocovariance matrix

(4) $${\mathrm{Cov}}_{\tau }\left(x\right)=E\;\left(\left(x_{t}-\mu \right){\left(x_{t+\tau }-\mu \right)}^{\prime }\right)$$

with a given lag *τ* > 0 to solve the SOS problem. An orthogonal matrix ***U*** = (***u***_1_, …, ***u***_*p*_)^′^ is found simply using the eigendecomposition of the autocovariance matrix as, by assumption, ${\mathrm{Cov}}_{\tau }\left(x_{t}^{\mathrm{st}}\right)=U\;{\mathrm{Cov}}_{\tau }\left(z\right)U^{\prime }={\mathrm{UD}}_{\tau }U^{\prime }$ for any lag *τ*. Equivalently, ***U*** is found by maximizing

(5) $$∥\mathrm{diag}\left(U\;{\mathrm{Cov}}_{\tau }\left(x_{t}^{\mathrm{st}}\right)U^{\prime }\right){∥}^{2}=\sum_{i=1}^{p}{\left(u_{i}^{\prime }\;{\mathrm{Cov}}_{\tau }\left(x_{t}^{\mathrm{st}}\right)u_{i}\right)}^{2}$$

for a given lag *τ*, under the orthogonality constraint ***UU*** ^′^ = ***I***_*p*_. Here ∥ ⋅ ∥ is the matrix (Frobenius) norm. Notice that the signal separation matrix ***W*** = ***U*** Cov (***x***_*t*_)^−1/2^ is uniquely defined only if all the eigenvalues of ${\mathrm{Cov}}_{\tau }\left(x_{t}^{\mathrm{st}}\right)$ are distinct. The signal separation matrix ***W*** is affine equivariant in the sense that if $x_{t}^{*}={\mathrm{Bx}}_{t}+b$ for all nonsingular ***B*** ∈ ℝ^*p* × *p*^ and for all vectors ***b*** ∈ ℝ^*p*^, then the signal separation matrix based on $x_{t}^{*}$ is ***W*** ^*^ = ***WB*** ^−1^ up to sign changes of the components (Miettinen et al., 2012). In practice, affine equivariance means that the separation result does not depend on the mixing procedure. This is a desirable property for a BSS method.

The main shortcoming of the AMUSE method is that it uses only one cross‐autocovariance matrix for signal separation. Its performance thus depends heavily on the chosen lag *τ*. There are several extensions to AMUSE found in the literature which aim at better separation results (see, e.g., Chapter 7 of Comon & Jutten, 2010). One extension of AMUSE has become recently very popular. Belouchrani et al. (1997) introduced the SOBI method that jointly diagonalizes several autocovariance matrices simultaneously. Like AMUSE, the SOBI signal separation matrix is also affine equivariant. Notice that as more than two matrices can be exactly jointly diagonalized only if they commute with each other, as is the case here at the population level, approximate joint diagonalization of the autocovariance matrices is needed in practice as the matrices are estimated with an error. The solution for the signal separation matrix ***W*** in SOBI is thus achieved by formulating the maximization problem

(6) $$\sum_{\tau \in T}∥\mathrm{diag}\left(U\;{\mathrm{Cov}}_{\tau }\left(x_{t}^{\mathrm{st}}\right)U^{\prime }\right){∥}^{2}=\sum_{\tau \in T}\sum_{i=1}^{p}{\left(u_{i}^{\prime }\;{\mathrm{Cov}}_{\tau }\left(x_{t}^{\mathrm{st}}\right)u_{i}\right)}^{2},$$

for an orthogonal matrix ***U***, where $T=\left\{\tau_{1},\ldots ,\tau_{K}\right\}\subset {\mathbb{Z}}_{+}$ is a set of selected lags. As before, the SOBI signal separation matrix is given by ***W*** = ***U*** Cov (***x***_*t*_)^−1/2^. In symmetric SOBI the rows of ***U*** are found simultaneously (Miettinen et al., 2016), and the most efficient algorithm for such simultaneous joint diagonalization is based on Jacobi rotations (Clarkson, 1988). In deflation‐based SOBI the uncorrelated components are found one by one (Miettinen et al., 2014). These two approaches are discussed and compared thoroughly in Illner et al. (2015) and in Miettinen et al. (2016). In SOBI, we use (6) as a criterion for joint diagonalization. For other proposals for joint diagonalization criteria, see Ziehe et al. (2003); Yeredor (2000); Pham (2001), for example. Yeredor (2002) suggests an algorithm (Alternating Columns—Diagonal Centers, ACDC), where the diagonalizer does not have to be an orthogonal matrix. For a summary of joint diagonalization methods used in blind signal separation, see also Theis and Inouye (2006).

The AMUSE and SOBI estimates for ***W*** ∈ ℝ^*p* × *p*^ are obtained by replacing ***μ***, Cov (***x***_*t*_) and Cov_*τ*_(***x***_*t*_) in the estimation procedure by the sample mean vector $\bar{x}$, the sample covariance matrix $\hat{\;\mathrm{Cov}\;}\left(x_{t}\right)$ and the symmetrized autocovariance matrix

(7) $${\hat{\;\mathrm{Cov}\;}}_{\tau }^{S}\left(x_{t}\right)=\frac{1}{2}\left({\hat{\;\mathrm{Cov}}}_{\tau }\left(x_{t}\right)+{\hat{\mathrm{Cov}}}_{\tau }{\left(x_{t}\right)}^{\prime }\right),$$

where

$${\hat{\;\mathrm{Cov}}}_{\tau }\left(x\right)=\frac{1}{T-\tau }\sum_{t=1}^{T-\tau }\;x_{t}x_{t-\tau }^{\prime },\;\tau =0,1,2,\ldots ,$$

respectively. The estimates of $\hat{U}$ are then obtained using (5) and (6) for AMUSE and SOBI, respectively, and the signal separation matrix estimates are obtained as $\hat{W}=\hat{U}\hat{\;\mathrm{Cov}\;}{\left(x_{t}\right)}^{-1/2}$.

In Miettinen et al. (2012, 2014, 2016) it was shown that, for both AMUSE and SOBI methods, the joint limiting distribution of a signal separation matrix $\hat{W}$, based on an observed time series ***x***_1_, …, ***x***_*T*_, depends on the joint limiting distribution of the sample covariance matrix and the symmetrized autocovariance matrices. Limiting distributions in case of multivariate MA(∞) processes are considered as special case. Consider now the model (1) with ***μ*** = **0** (wlog). We assume that ***z***_*t*_ is a multivariate MA(∞) process that fulfills the assumptions (2) and (3). We also assume that the components of ***z***_*t*_ have finite fourth moments and they are exchangeable and marginally symmetric, that is, ***JPz***_*t*_ ∼ ***z***_*t*_, for all sign change matrices ***J*** and permutation matrices ***P***. As AMUSE and SOBI estimates $\hat{W}$ are affine equivariant, we can assume that ***W*** = ***I***_*p*_. Thus ${\mathrm{Cov}}_{\tau }^{S}\left(z_{t}\right)=D_{\tau }$, in which the diagonal elements are distinct and in decreasing order. The joint limiting distribution of

$$\sqrt{T}\left(\begin{array}{c} \mathrm{vec}\left(\hat{\mathrm{Cov}\;}\left(z_{t}\right)-I_{p}\right)\\ \mathrm{vec}\left({\hat{\mathrm{Cov}}}_{\tau_{1}}^{S}\left(z_{t}\right)-D_{\tau_{1}}\right)\\ \vdots \\ \mathrm{vec}\left({\hat{\mathrm{Cov}}}_{\tau_{K}}^{S}\left(z_{t}\right)-D_{\tau_{K}}\right) \end{array}\right)$$

is then a (*K* + 1)*p* ^2^‐variate normal distribution with mean value zero and covariance matrix as given in Miettinen et al. (2016), and the limiting distribution of

$$\sqrt{T}\;\mathrm{vec}\left(\hat{W}-I_{p}\right)$$

is a *p* ^2^‐variate normal distribution with mean value zero and limiting covariance matrices as given in Miettinen et al. (2012, 2014, 2016). Asymptotic and final sample efficiency studies demonstrate that in most settings the symmetric SOBI estimate outperforms both the AMUSE estimate and the deflation‐based SOBI estimate.

As mentioned earlier, the goal of SOBI is to incorporate the information of more lags as compared to AMUSE which is based on a single lag. SOBI does so by generalizing the idea of the diagonalization of two matrices to jointly diagonalizing more matrices. But there are other ways to include the information of more lags. For example, temporal decorrelation source separation or time‐delays based separation (TDSEP, Ziehe & Müller, 1998) is a BSS algorithm which generalizes AMUSE in another direction. One version of the algorithm uses the average of several autocovariance matrices, that is,

(8) $$\frac{1}{|T|}\sum_{\tau \in T}\;{\mathrm{Cov}}_{\tau }\left(x_{t}^{\mathrm{st}}\right)$$

to find the unknown rotation matrix. Thus, such a TDSEP algorithm can be viewed as an extension of AMUSE, where ${\mathrm{Cov}}_{\tau }\left(x_{t}^{\mathrm{st}}\right)$ for a lag *τ* is replaced by (8). It seems, however, that SOBI prevails in practical applications as compared to TDSEP.

Finally note that Belouchrani and Amin (1998) considered blind source separation in the frequency domain. Instead of joint diagonalizing covariance matrices, their time‐frequency separation (TFS) algorithm jointly diagonalizes spatial time‐frequency distribution matrices of the form

(9) $$D_{\mathrm{zz}}\left(t,f\right)=\sum_{l=-\infty }^{\infty }\sum_{m=-\infty }^{\infty }\phi \left(m,l\right)z_{t+m+l}z_{t+m-l}^{\prime }e^{j4\mathrm{\pi fl}},$$

where the kernel *ϕ*(*m*, *l*) characterizes the distribution, *t* refers to time and *f* to frequency (Cohen, 1995).

## 3 GENERALIZATIONS AND OTHER VERSIONS OF SOBI

SOBI has proven to be a simple and powerful method for time series dimension reduction. In some settings its applicability is, however, limited, therefore several generalizations and modifications of the method have been proposed in the literature. The generalizations of SOBI try to answer some of the issues the regular SOBI has:

- There are no tools for selecting a lag set that yields best separation performance.
- Exact diagonalization of the covariance matrix may happen at the expense of poorer diagonalization of the autocovariance matrices.
- The method does not work well when the volatility cannot be considered as fixed throughout the time series.
- The mixing procedure is assumed to stay constant all the time.
- The latent components are assumed to be weakly stationary.
- The method can only be used for vector‐valued time series, but not for images or tensor‐valued time series.
- The method is not robust to outliers.
- The method does not give a way to directly assess which of the signals are important.
- The method is not applicable if there are more sources than observed signals.

In the following subsections, we introduce some extensions of AMUSE and SOBI which address the restrictions expressed above. Table 1 summarizes these extensions and describes assumptions needed for each method.

**TABLE 1:** Overview of SOBI and its variants considered in this review and assumptions needed for each method

Method	Data assumptions	Reference(s)
AMUSE, SOBI, TDSEP	Uncorrelated weakly stationary processes where most information is in the second moments. Constant mixing matrix	Tong et al. (1990), Belouchrani et al. (1997), Ziehe and Müller (1998)
aSOBI, eSOBI	Sources are uncorrelated stationary *MA*(∞)‐processes. Constant mixing matrix	Miettinen (2015), Taskinen et al. (2016)
WASOBI	Uncorrelated weakly stationary processes where most information is in the second moments with some additional information on the sources available. Constant mixing matrix	Yeredor (2000)
vSOBI, FixNA, FixNA2	Independent stationary processes where most information is in the higher order moments, for example, stochastic volatility models	Matilainen et al. (2017), Shi et al. (2009), Hyvärinen (2001)
gSOBI	Convex linear combination of SOBI and vSOBI assuming independent stationary processes with information in second and/or higher order moments	Miettinen, Matilainen, et al. (2020)
TV‐SOBI	Uncorrelated weakly stationary processes where most information is in the second moments. Mixing matrix changes over time	Yeredor (2003), Weisman and Yeredor (2006)
NSS‐TD‐JD	*L* independent *p*‐variate time series with uncorrelated weakly stationary sources and all having the same mixing matrix or block‐stationary *p*‐variate time series where the sources have constant mean but variances and covariance functions of them are block‐stationary	Choi and Cichocki (2000)
mdSOBI	Uncorrelated multidimensional data such as images which are weakly stationary	Theis et al. (2004)
TSOBI	Tensorial time series with uncorrelated weakly stationary sources	Virta and Nordhausen (2017b)
SOBIUM	Uncorrelated weakly stationary processes. The number of sources exceeds the number of observed components	Lathauwer and Castaing (2008)
SAM‐SOBI, eSAM‐SOBI	Robust variants of SOBI which make the additional assumption of symmetric sources	Theis et al. (2010), Ilmonen et al. (2015)
RmdSOBI	Robust variant of mdSOBI which makes the additional assumption of symmetric sources	Lietzén et al. (2017)

### 3.1 Using asymptotics for gaining better separation

The signal separation performance of SOBI method depends heavily on the chosen lag set $T=\left\{\tau_{1},\ldots ,\tau_{K}\right\}$. A default lag set in many applications has been {1, …, 12}, as these lags are thought to be enough for capturing the correlation structure in data. However, this is just an arbitrary choice, and there are no theoretical foundations for choosing the set. Tang, Liu, and Sutherland (2005), for example, advocate for EEG that the default set is not sufficient.

Miettinen (2015) and Taskinen et al. (2016) have proposed methods that use asymptotic results to obtain more efficient signal separation matrix estimates. Note first that the sum of the limiting variances of the off‐diagonal elements of $\sqrt{T}\mathrm{vec}\left(\hat{W}-I_{p}\right)$ provides a global measure of the variation of $\hat{W}$ and thus a tool for comparing asymptotic efficiencies (Ilmonen et al., 2010,b). Miettinen (2015) proposed a method, where the criterion function to be maximized is

$$\sum_{\tau \in T}\sum_{i=1}^{p}G\left(u_{i}^{\prime }\;{\mathrm{Cov}}_{\tau }\left(x_{t}^{\mathrm{st}}\right)u_{i}\right),$$

where *G* is an even and continuously differentiable function, for which *G*(0) = 0, *g*(*x*) = *G* ^′^(*x*) ≥ 0 for *x* > 0 and *g*(*x*) ≤ 0 for *x* < 0. The choice *G*(*x*) = *x* ^2^ gives the regular SOBI estimator. Also asymptotic properties of this estimate have been carefully investigated. Miettinen (2015) focused especially on aSOBI, a version of the symmetric SOBI method, that uses

(10) $$\sum_{\tau \in T}\sum_{i=1}^{p}{\left|u_{i}^{\prime }\;{\mathrm{Cov}}_{\tau }\left(x_{t}^{\mathrm{st}}\right)u_{i}\right|}^{a},$$

where *a* ∈ (1, ∞), as a criterion function. A large value of *a* means that more emphasis is put on the matrices with large autocovariances. An adaptive estimator based on aSOBI is suggested to improve efficiency of signal separation. First the regular SOBI is used to obtain estimates for source components. Then the asymptotic results are used to find the value *a* that minimizes the sum of the limiting variances. Finally, the signal separation matrix is estimated using the chosen *a* (Miettinen, 2015).

In Taskinen et al. (2016), similar approach as in aSOBI is used, but instead of using different *G*‐functions, the most efficient combination of lags is chosen. In efficient SOBI (eSOBI) one compiles a list of different lag combinations, and the lag set that leads to the lowest measure of variation is then chosen for the problem at hand. Notice that for computing limiting variances, both aSOBI and eSOBI assume that the signals are *MA*(∞) processes.

### 3.2 Reformulating SOBI as weighted least squares problem

Due to the prewhitening step in SOBI, the covariance matrix is exactly diagonalized while the autocovariance matrices are approximately jointly diagonalized. This exact diagonalization of the covariance matrix may, however, happen at the expense of poorer diagonalization of the autocovariance matrices (Cardoso, 1994). The intuition is that the global maximal diagonality of autocovariance matrices are most likely achieved when the covariance matrix is not exactly diagonal, but the diagonal covariance is mandatory for SOBI's joint diagonalization in (6). Also, when estimating the correlations, the errors are highly correlated. The use of a weighted least squares criterion can thus be beneficial when compared to the ordinary least squares optimization criterion that is used in classical SOBI.

Yeredor (2000) has suggested weights‐adjusted SOBI (WASOBI) algorithm, where instead of the approximate joint diagonalization, the problem is reformulated as a weighted least squares problem. Observe that

(11) $$\hat{y}=G_{0}\left(A\right)\mathrm{diag}\left(D\right),$$

which is a stacked form over all τ∈T of the following equation,

(12) $${\hat{y}}_{\tau }=G\left(A\right)\mathrm{diag}\left(D_{\tau }\right),$$

where ${\hat{y}}_{\tau }=\mathrm{vec}\left({\hat{\mathrm{Cov}}}_{\tau }\left(x_{t}^{\mathrm{st}}\right)\right)$, *G*(***A***) = ***A*** ⊙ ***A*** with ⊙ denoting Khatri–Rao product, and *G*_0_(***A***) = ***I***_*p*_ ⊗ *G*(***A***). Since the symmetry property cannot be ensured in the vectorized least squared estimation, in practice ${\hat{y}}_{\tau }$ is more commonly assigned as ${\hat{y}}_{\tau }=\text{svec}\left({\hat{\mathrm{Cov}}}_{\tau }\left(x_{t}^{\mathrm{st}}\right)\right)$, which is achieved by stacking only the lower triangle (including diagonal positions) of $\frac{1}{2}\left({\hat{\mathrm{Cov}}}_{\tau }\left(x_{t}^{\mathrm{st}}\right)+{\hat{\mathrm{Cov}}}_{\tau }^{\prime }\;\left(x_{t}^{\mathrm{st}}\right)\right)$. Meanwhile, the design matrix ***H*** containing 0, 1 and 1/2 is introduced for *G*_0_(***A***) = ***I***_*p*_ ⊗ (***H*** (***A*** ⊙ ***A***)) to conform matrix multiplication (Tichavský, Doron, et al., 2006). Finally, weighted least squares theory suggests that using $\hat{y}$ as in Equation (11), the following criterion should be minimized

(13) $$C_{\mathrm{WLS}}\left(A,\mathrm{diag}\left(D\right)\right)={\left(\hat{y}-G_{0}\left(A\right)\mathrm{diag}\left(D\right)\right)}^{\prime }Q\left(\hat{y}-G_{0}\left(A\right)\mathrm{diag}\left(D\right)\right),$$

where the appropriate weight ***Q*** is calculated based on the correlations between the (*i*, *j*)th element of ${\hat{\mathrm{Cov}}}_{\tau }\left(x_{t}^{\mathrm{st}}\right)$ and the (*k*, *l*)th element of ${\hat{\mathrm{Cov}}}_{\tau }\left(x_{t}^{\mathrm{st}}\right)$. The weighted least square problem is solved by an iterative manner. For convenience, write ***f*** (*θ*) = *G*_0_(***A***)diag(***D***), where ***θ*** is a vector containing unknown parameters in ***A*** and diag(***D***). The minimizing criteria (13) can then be written as $C_{\mathrm{WLS}}\left(\theta \right)={\left(\hat{y}-f\left(\theta \right)\right)}^{\prime }Q\left(\hat{y}-f\left(\theta \right)\right)$. The iteration step is thus defined by

(14) $$\theta^{\left[i+1\right]}=\theta^{\left[i\right]}+{\left(F_{i}^{\prime }\;{\mathrm{QF}}_{i}\right)}^{-1}{\mathrm{QF}}_{i}\left(\hat{y}-f\left(\theta^{\left[i\right]}\right)\right),$$

where ${\left(F_{i}=\partial f\left(\theta \right)/\partial \theta \right|}_{\theta =\theta^{\left[i\right]}}$ (Tichavský, Doron, et al., 2006).

The computational complexity of WASOBI can be relaxed when the sources are assumed to follow a known time series model. Optimal weighting for moving average sources was presented in Yeredor (2000), whereas AR‐WASOBI is a fast algorithm for autoregressive sources (Tichavský, Doron, et al., 2006; Tichavský & Yeredor, 2009). Tichavský, Koldovský, et al. (2006) have also proposed EFWS (EFica‐WaSobi) and COMBI (COMBInation), which are both combinations of the efficient variant of fastICA (EFICA) (Koldovský et al., 2006) and WASOBI. Other methods, which do not require exact diagonalization of the covariance matrix are proposed in Pham (2001); Yeredor (2002), for example.

### 3.3 Separating time series with volatility clustering

Regular SOBI methods do not work if the time series exhibit volatility clustering, that is, in cases where the volatility cannot be considered constant over time. Examples of such times series models include stochastic volatility (SV) models and generalized autoregressive conditional heteroskedasticity (GARCH) models (Bollerslev, 1986; Taylor, 1982). See also Matteson and Tsay (2011) for a recent review on GARCH models. For such models the autocovariances (3) equal to zero. However, zero autocovariances do not imply the absence of temporal dependence.

In Matilainen et al. (2017) the SOBI method is extended to the case of volatility clustering. Notice first that the maximization problem in SOBI (6) can alternatively be written as

(15) $$\sum_{\tau \in T}\sum_{i=1}^{p}{\left(\;E\;\left(\left(u_{i}^{\prime }x_{t}^{\mathrm{st}}\right)\left(u_{i}^{\prime }x_{t+\tau }^{\mathrm{st}}\right)\right)\right)}^{2}.$$

A set of methods suitable for times series exhibiting volatility clustering is then obtained by using nonlinearity functions in (15). In a variant of SOBI (vSOBI) proposed by Matilainen et al. (2017), the maximization problem for an orthogonal matrix ***U*** is

(16) $$\sum_{\tau \in T}\sum_{i=1}^{p}{\left(E\;\left(G\left(u_{i}^{\prime }x_{t+\tau }^{\mathrm{st}}\right)G\left(u_{i}^{\prime }x_{t}^{\mathrm{st}}\right)\right)-E\;\left(G\left(u_{i}^{\prime }x_{t}^{\mathrm{st}}\right)\right)\;E\;\left(G\left(u_{i}^{\prime }x_{t+\tau }^{\mathrm{st}}\right)\right)\right)}^{2},$$

where *G* is any twice continuously differentiable function. Some commonly used choices are *G*(*x*) = *x* ^2^ and *G*(*x*) = log(cosh(*x*)). Notice that the vSOBI method needs stronger assumptions than SOBI, as the components of ***z*** are required to be mutually independent, not just uncorrelated. The methods FixNA (Shi et al., 2009) and FixNA2 (Hyvärinen, 2001) are closely related to vSOBI. For the statistical properties and comparisons on these methods, see Matilainen et al. (2017). Dynamic orthogonal analysis (DOC), as suggested in Matteson and Tsay (2011), is very similar to vSOBI. Generalized fourth order blind identification (gFOBI) and generalized joint approximation of eigenmatrices (gJADE), as suggested in Matilainen et al. (2015), are designed for time series considered in DOC.

SOBI is thus applicable when variances of the source time series can be regarded as constant over time and vSOBI works for time series exhibiting volatility clustering. Miettinen, Matilainen, et al. (2020) combined SOBI and vSOBI with *G*(*z*) = *z* ^2^ and proposed a generalized SOBI (gSOBI) method, which maximizes

(17) $$\begin{array}{l} w\sum_{\tau \in T_{1}}\sum_{i=1}^{p}{\left(\;E\;\left(u_{i}^{\prime }x_{t}^{\mathrm{st}}u_{i}^{\prime }x_{t+\tau }^{\mathrm{st}}\right)\right)}^{2}+\left(1-w\right)\sum_{\tau \in T_{2}}\sum_{i=1}^{p}{\left(\;E\;\left({\left(u_{i}^{\prime }x_{t}^{\mathrm{st}}\right)}^{2}{\left(u_{i}^{\prime }x_{t+\tau }^{\mathrm{st}}\right)}^{2}-1\right)\right)}^{2}, \end{array}$$

under the constraint ***UU*** ^′^ = ***I***_*p*_. Here $T_{1}$ and $T_{2}$ are the sets of lags for the linear and quadratic parts, respectively, and 0 ≤ *w* ≤ 1 is a parameter which gives the weight for the two parts.

In Miettinen, Matilainen, et al. (2020) the limiting distribution of the signal separation matrix is derived under general conditions, and the asymptotic variances are derived in the case of ARMA‐GARCH model. Asymptotic and finite sample efficiency studies are used to compare different choices of a weight coefficient. As it is often of interest to identify all those components which exhibit stochastic volatility features, a method for ordering the time series according to their “volatility” is proposed along with the test statistics for testing linear autocorrelation and volatility clustering. Visual analytic tools which might help to choose *w*, $T_{1}$ and $T_{2}$ are suggested in Piccolotto et al. (2020).

### 3.4 Time‐dependent mixing matrix

When using SOBI, we assume a SOS model (1), where the mixing matrix ***A*** is assumed to be constant through time. However, we can also allow the mixing matrix to change over time. This enables the use of SOBI type methods also in cases where the observed signals are nonstationary, as the sources are still assumed to be stationary. Yeredor (2003) proposed time‐variable SOBI (TV‐SOBI) for such situations. The time‐variable BSS (TV‐BSS) model is written as

$$x_{t}=\mu +A_{t}z_{t},\;t\in \mathbb{Z},$$

where ***μ*** ∈ ℝ^*p*^ is again a location vector and ***A***_*t*_ ∈ ℝ^*p* × *p*^ is the mixing matrix at time *t*. The assumptions on ***z*** are the same as for SOBI. Yeredor (2003) concentrated on a case where the variation is assumed to be linear in time, that is,

(18) $$x_{t}=\mu +\left(I_{p}+tR\right)A_{0}z_{t},\;t\in \mathbb{Z},$$

where ***A***_0_ ∈ ℝ^*p* × *p*^ is a full‐rank mixing matrix at time zero and ***R*** ∈ ℝ^*p* × *p*^ is a “relative rate” matrix, which generates the time effect. In general, the values in matrix ***R*** are assumed to be very small, that is, the mixing matrix changes only a little at a time. Weisman and Yeredor (2006) considered models for periodical time variation, that is,

(19) $$x_{t}=\mu +\left(I_{p}+R_{c}\cos\left(\mathrm{\omega t}\right)+R_{s}\sin\left(\mathrm{\omega t}\right)\right)A_{m}z_{t},\;t\in \mathbb{Z},$$

where *ω* is an angular frequency, ***A***_*m*_ ∈ ℝ^*p* × *p*^ is a full‐rank matrix representing the “mean” mixing matrix, and matrices ***R***_*c*_, ***R***_*s*_ ∈ ℝ^*p* × *p*^ describe “relative rates” of harmonics generating periodical variation.

By definition, time variation increases complexity of the signal separation matrix estimation due to the increased number of parameters in models. The developed estimation methods involve standard SOBI procedures on top of applicable matrix algebra (Yeredor, 2003). In return, these approaches can outperform SOBI especially if the time series length *T* is very large and/or observation indicates clear periodic pattern. For more details on time‐varying mixtures in SOS, see, for example, Chapter 7 of Comon and Jutten (2010) and the references therein.

### 3.5 Multisubject SOS and block‐stationary SOS

So far it is assumed that the latent sources are stationary. This is a convenient but strong assumption, hence there are approaches which try to relax it. One motivation for such approaches are for example multisubject studies. Assume that we observe for *L* subjects *p*‐variate centered time series $x_{t}^{i}$ of length *T*_*i*_, *i* = 1, …, *L*. A popular model for example in neurosciences states that for all *L* subjects a SOS model holds with the additional assumption that the mixing matrix ***A*** is the same for all subjects. The latent components $z_{t}^{i}$ might, however, follow different processes. This is often referred to as group ICA, see, for example, Cong et al. (2013) and references therein.

One way to analyze multisubject data is to concatenate the time series into $x_{t^{\prime }}=\left(x_{t}^{1},\ldots ,x_{t}^{L}\right)$ with $t^{\prime }=1,\ldots ,\sum_{l=1}^{L}T_{l}$, and to apply a BSS method to $x_{t^{\prime }}$. Naturally $x_{t^{\prime }}$ is not anymore stationary, but rather block‐stationary, where the observations coming from the same subject form blocks. This information is exploited in a block‐stationary variant of SOBI known as nonstationary source separation with temporal delayed correlation matrices using joint diagonalization (NSS‐TD‐JD, Choi and Cichocki (2000)). For that purpose, define the block autocovariance matrix as

$${\hat{\;\mathrm{Cov}\;}}_{\tau }^{l}\left(x_{t^{\prime }}\right)=\frac{1}{T_{l}-\tau }\sum_{t^{\prime }\in t_{l}}\;x_{t^{\prime }+\tau }x_{t^{\prime }}^{\prime },$$

where *t*_*l*_ indicates the index in *t* ^′^ which identifies block *l*, *l* = 1, …, *L*. In NSS‐TD‐JD the lag set $T=\left\{0,\tau_{1},\ldots ,\tau_{K}\right\}$ contains usually the zero lag, and the unmixing matrix is obtained using the following steps. Write the standardized times series as $x_{t^{\prime }}^{\mathrm{st}}=\hat{\;\mathrm{Cov}\;}{\left(x_{t^{\prime }}\right)}^{-1/2}\left(x_{t^{\prime }}-{\bar{x}}_{t^{\prime }}\right)$ and find the orthogonal matrix **U** maximizing

$$\sum_{l\in L}\sum_{\tau \in T}{\left\|U{\hat{\mathrm{Cov}\;}}_{\tau }^{l}\left(x_{t^{\prime }}\right)U^{\prime }\right\|}^{2}.$$

The signal separating matrix is then $W=U\hat{\mathrm{Cov}\;}{\left(x_{t^{\prime }}\right)}^{-1/2}$. Notice that there are in total *L*(*K* + 1) matrices to be jointly diagonalized. If *L* = 1, the method just reduces to SOBI.

In a group ICA context the number of blocks and their ranges are naturally given. In nonstationary source separation (NSS) it is assumed that $z_{t^{\prime }}$ is first‐order stationary and that ${\mathrm{Cov}}_{\tau }\left(z_{t^{\prime }}\right)=D_{\tau ,t^{\prime }}$ is a diagonal matrix dependent on *τ* and *t* ^′^. NSS‐TD‐JD was originally suggested in this setting and requires dividing the process in *L* blocks. Hence *L* and *K* are parameters which have impact on the performance of the method.

### 3.6 Generalization to *m*‐dimensional data

**FIGURE 1:**
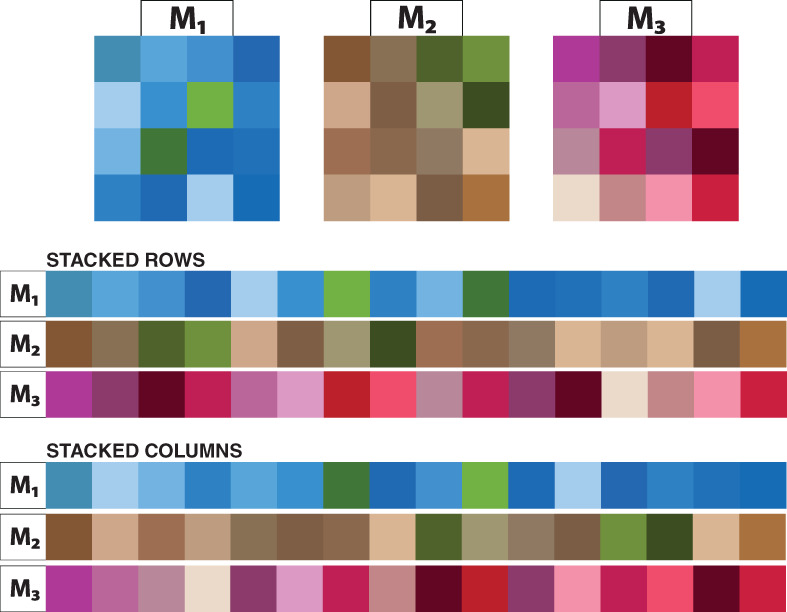
Schematic representation of the difference when stacking rows and columns respectively

Demixing *p* images is a frequent application area of BSS. It is obvious that in image data there is serial dependence present within columns and rows of the images although one usually assumes more general local spatial dependencies. A common, though naive, way to approach this problem is to vectorize the images and use these vectors as input in standard BSS methods such as AMUSE or SOBI. The separation result then depends of course on whether the rows or columns have been stacked as for example visualized in Figure 1. Also, an image may actually be represented not as a 2‐dimensional object, but for example as a 3‐dimensional object as, besides width and height, also the intensity of the colors is measured. Thus, images can be thought of as *p m*‐dimensional objects of the same size or as *p*‐dimensional vectors with a *m*‐dimensional index. Using the later representation Theis et al. (2004) suggested the concept of multidimensional autocovariance, which is defined for a centered *p*‐variate vector ***x***_***t***_ with an *m*‐dimensional index ***t*** = (*t*_1_, …, *t*_*m*_) as

$${\mathrm{Cov}}_{\tau }\left(x_{t}\right)=E\left(x_{t}x_{t+\tau }^{\prime }\right)=E\left(x_{t_{1},\ldots ,t_{m}}x_{t_{1}+\tau_{1},\ldots ,t_{m}+\tau_{m}}^{\prime }\right),$$

where *τ* = (*τ*_1_, …, *τ*_*m*_) specifies the lags for the corresponding dimensions.

Replacing regular symmetrized autocovariance matrices (7) with multidimensional autocovariance matrices and their symmetrized versions in the regular AMUSE and SOBI yields what Theis et al. (2004) refer to as multidimensional AMUSE (mdAMUSE) and multidimensional SOBI (mdSOBI), respectively, which they then apply for example to fMRI data.

**FIGURE 2:**
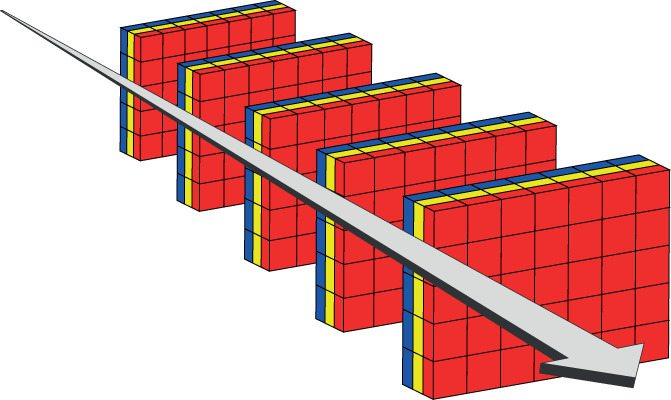
Representation of a time series of mode three tensors

In mdAMUSE and mdSOBI we assume that *p* multidimensional objects are observed once. However, one can also observe a series of multidimension objects, so‐called tensorial time series. In tensorial time series at each time point *t* a *m*‐dimensional tensor $X_{t}\in {\mathbb{R}}^{p_{1}\times \cdots \times p_{m}}$ is observed. Videos serve as examples of typical tensorial time series, where for each frame a tensor is observed, see Figure 2 for a visualization. In case of a black and white video *m* = 2 and in case of a colored video *m* = 3 as indicated above. Tensorial AMUSE (TAMUSE) and tensorial SOBI (TSOBI) were introduced in Virta and Nordhausen (2017b) and are defined for any positive integer *m*, where with *m* = 1 the standard methods for vector‐valued data are obtained. Consider next the case *m* = 2, that is, at each time point a matrix ***X***_*t*_ is observed.

The corresponding model for tensorial, that is, matrix‐variate SOS is

$$X_{t}=A_{L}Z_{t}A_{R}^{\prime }+\mu ,$$

where $\mu \in {\mathbb{R}}^{p_{1}\times p_{2}}$ specifies the location, $A_{L}\in {\mathbb{R}}^{p_{1}\times p_{1}}$ and $A_{R}\in {\mathbb{R}}^{p_{2}\times p_{2}}$ are the nonsingular left and right mixing matrices describing the induced column and row dependencies on the latent process $Z_{t}\in {\mathbb{R}}^{p_{1}\times p_{2}}$ which fulfills the assumptions

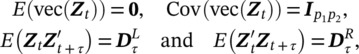

where $D_{\tau }^{L}$ and $D_{\tau }^{R}$ are diagonal for all *τ* = 1, 2, …. Thus, the assumptions are a straightforward generalizations of the assumptions (2) and (3) listed in the vector‐valued case.

Virta and Nordhausen (2017b) showed that the unmixing can be done mode‐wise, where the left and right covariance matrices needed for whitening are

$${\mathrm{Cov}}_{L}\left(X_{t}\right)=\frac{1}{p_{2}}E\left(\left(X_{t}-{\bar{X}}_{t}\right){\left(X_{t}-{\bar{X}}_{t}\right)}^{\prime }\right)$$

and

$${\mathrm{Cov}}_{R}\left(X_{t}\right)=\frac{1}{p_{1}}E\left({\left(X_{t}-{\bar{X}}_{t}\right)}^{\prime }\left(X_{t}-{\bar{X}}_{t}\right)\right),$$

yielding the standardized matrix‐valued time series

$$X_{t}^{\mathrm{st}}={\mathrm{Cov}}_{L}{\left(X_{t}\right)}^{-1/2}\left(X_{t}-{\bar{X}}_{t}\right)\;{\mathrm{Cov}}_{R}{\left(X_{t}\right)}^{-1/2}.$$

Accordingly, the mode‐wise autocovariance matrices with lag *τ* are defined as

$${\mathrm{Cov}}_{L,\tau }\left(X_{t}\right)=\frac{1}{p_{2}}E\left(\left(X_{t}-{\bar{X}}_{t}\right){\left(X_{t+\tau }-{\bar{X}}_{t}\right)}^{\prime }\right)$$

and

$${\mathrm{Cov}}_{R,\tau }\left(X_{t}\right)=\frac{1}{p_{1}}E\left({\left(X_{t}-{\bar{X}}_{t}\right)}^{\prime }\left(X_{t+\tau }-{\bar{X}}_{t}\right)\right),$$

which can then be used to find the left and right rotation matrices ***U***_*L*_ and ***U***_*R*_ as the maximizers of

$$\sum_{\tau \in T_{L}}∥\mathrm{diag}\left(U\;{\mathrm{Cov}}_{L,\tau }\left(X_{t}^{\mathrm{st}}\right)U^{\prime }\right){∥}^{2}\;\text{and}\;\sum_{\tau \in T_{R}}∥\mathrm{diag}\left(U\;{\mathrm{Cov}}_{R,\tau }\left(X_{t}^{\mathrm{st}}\right)U^{\prime }\right){∥}^{2},$$

respectively, yielding ***W***_*L*_ = ***U***_*L*_ Cov_*L*_(***X***_*t*_)^−1/2^ and ***W***_*R*_ = ***U***_*R*_ Cov_*R*_(***X***_*t*_)^−1/2^. Depending on the number of lags used in $T_{L}$ and $T_{R}$ one distinguishes between tensorial AMUSE or tensorial SOBI.

The extension to higher order tensors follows the same ideas as in the matrix case and the key is still to unmix modewise. Actually, when considering the *m*th mode of the tensor $X\in {\mathbb{R}}^{p_{1}\times \cdots \times p_{r}}$, the tensor is flattened or matricized to its *m*‐flattening matrix $X^{\left(m\right)}\in {\mathbb{R}}^{p_{m}\times \rho_{m}}$ horizontally by stacking all its *m*‐mode vectors where accordingly $\rho_{m}=\prod_{i\neq m}^{r}p_{i}$. To simplify in the following the notation we define the multiplication operator ⊙_*m*_ over a tensor $X\in {\mathbb{R}}^{p_{1}\times \cdots \times p_{r}}$ and a matrix $A\in {\mathbb{R}}^{p_{m}\times p_{m}}$ in an element‐wise manner as,

$${\left(X{⊙}_{m}A\right)}_{i_{1}\cdots i_{r}}=\sum_{j_{m}}x_{i_{1}\cdots i_{m-1}j_{m}i_{m+1}\cdots i_{r}}a_{i_{m}j_{m}},$$

where *m* = 1, 2, …, *r* and the product $\left(X{⊙}_{m}A\right)$ has the same dimensionality as X. The tensor blind source separation model for a tensor‐valued time series $X_{t}$ can then be written as

$$X_{t}={\mathbb{Z}}_{t}{⊙}_{1}A_{1}\cdots {⊙}_{r}A_{r},$$

where $A_{m}\in {\mathbb{R}}^{p_{m}\times p_{m}}$ are *m*‐mode mixing matrices, and the unobserved tensor‐valued ℤ_*t*_ fulfills the assumptions

$$E\;\left[\mathrm{vec}\left({\mathbb{Z}}_{t}\right)\right]=0,\;\mathrm{Cov}\;\left[\mathrm{vec}\left({\mathbb{Z}}_{t}\right)\right]=I\;\text{and}\;E\;\left[{\mathbb{Z}}_{t}^{\left(m\right)}{\mathbb{Z}}_{t+\tau }^{{\left(m\right)}^{\prime }}\right]=E\;\left[{\mathbb{Z}}_{t+\tau }^{\left(m\right)}{\mathbb{Z}}_{t}^{{\left(m\right)}^{\prime }}\right]=D_{\tau }^{m},$$

which means that again, as in the vector and matrix case, the matrices $D_{\tau }^{m}$ are required to be diagonal for all modes *m* = 1, …, *r* and all lags *τ*. Assume for simplicity that in the following $X_{t}$ is centered, then the *m*‐mode (auto) covariance matrix is defined as

$${\mathrm{Cov}}_{\tau }^{m}\left(X_{t}\right)=\frac{1}{\rho_{m}}\;E\;\left(X_{t}^{\left(m\right)}{\left(X_{t+\tau }^{\left(m\right)}\right)}^{\prime }\right)$$

and accordingly the tensor is standardized from each mode

$$X_{t}^{\mathrm{st}}=X_{t}{⊙}_{1}{\left({\mathrm{Cov}}_{0}^{1}\left(X_{t}\right)\right)}^{-1/2}\cdots {⊙}_{r}{\left(\;{\mathrm{Cov}}_{0}^{r}\left(X_{t}\right)\right)}^{-1/2}.$$

Virta and Nordhausen (2017b) then show that subsequently the *m*th mode unmixing matrix is $W^{m}\left(X_{t}\right)=U_{m}^{\prime }{\left({\mathrm{Cov}}_{0}^{m}\left(X_{t}\right)\right)}^{-1/2}$ where ***U***_*m*_ is the orthogonal matrix which maximizes

$$\sum_{\tau \in T^{m}}∥\mathrm{diag}(U\;{\mathrm{Cov}}_{\tau }^{m}\left(X_{t}^{\mathrm{st}}\right)U^{\prime }{∥}^{2}.$$

Extensions to tensorial nonstationary time series following the same ideas are discussed in Virta and Nordhausen (2017a).

### 3.7 Robust SOBI methods

The sample statistics used in SOBI and most of the related methods, that is, the sample mean, the sample covariance matrix and the sample cross‐autocovariance matrix are highly sensitive to outlying observations and inefficient under heavy‐tailed distributions. To overcome this issue some robust counterparts for classical SOBI have been derived in the literature. Belouchrani and Cichocki (2000) proposed a robust standardization step for the SOBI procedure by applying in standardization a linear combination of selected autocovariance matrices, that is ${\left(\sum_{\tau \in T}\alpha_{\tau }\;{\mathrm{Cov}}_{\tau }\left(x_{t}\right)\right)}^{-1/2}$, where the parameters ***α***_*τ*_ are determined in an iterative method that ensure the positive‐definite property. The existence of ***α***_*τ*_ is proved by Tong et al. (1992). Notice, however, that for deriving genuine robust unmixing matrix estimates, we need robust estimates of location, covariance matrix and autocovariance matrix which measure the same quantity as their nonrobust counterparts and satisfy assumptions (2) and (3), that is, the covariance matrices are diagonal under the SOS model. This usually requires assuming symmetric components Nordhausen and Tyler (2015).

Theis et al. (2010) proposed a sign autocovariance SOBI (SAM‐SOBI) algorithm, where data are first centered as

(20) $$x_{t}^{\mathrm{st}}=\text{SCov}\;{\left(x_{t}\right)}^{-1/2}\left(x_{t}-\mu_{S}\right),$$

where ***μ***_*S*_ is the spatial median (see, e.g., Haldane, 1948) and SCov (***x***) = E (∥ ***x*** ∥^−2^ ***xx*** ^′^), where ∥ ***x*** ∥  = (***x*** ^′^ ***x***)^1/2^, is the spatial sign covariance matrix (Visuri et al., 2000). The spatial sign autocovariance matrices

$${\text{SCov}}_{\tau }=E\;\left(\frac{x_{t}}{∥x_{t}∥}\frac{x_{t+\tau }^{\prime }}{∥x_{t+\tau }∥}\right)$$

are then calculated for all lags τ∈T and symmetrized as in (7). Finally, a joint diagonalization of spatial sign autocovariance matrices is used to obtain an estimate for the signal separation matrix. The proposed method is highly robust. However, the obtained signal separation matrix estimate is not affine equivariant, as the spatial median and the spatial sign covariance matrix are only orthogonally equivariant (Visuri et al., 2000).

Ilmonen et al. (2015) proposed an eSAM‐SOBI method, which is an affine equivariant version of SAM‐SOBI. The spatial sign autocovariance matrices are still used in joint diagonalization, but the sample mean and the sample covariance matrix in (20) are replaced by the Hettmansperger–Randles estimates of location and scatter (Hettmansperger & Randles, 2002). These estimates are not only robust, but also affine equivariant. Simulation studies demonstrate that eSAM‐SOBI outperforms both SOBI and SAM‐SOBI under contaminated data. In Lietzén et al. (2017) RmdSOBI, a robust version of mdSOBI Theis et al. (2004), is given. Here whitening of the series is done using the robust Hettmansperger–Randles estimates of location and scatter, and multidimensional autocovariance matrices are replaced with the multidimensional spatial sign autocovariance matrices. Similarly, robust nonstationary blind source separation using spatial signs is discussed in Nordhausen (2014).

### 3.8 Choosing the dimension of the signal subspace

Most users of BSS methods assume that actually only a few of the latent components are of interest and the others are noise. For the differentiation of noise and signal “noisy” BSS models are needed. The two widely used models are the following. The external noise model (ENM) is given as

$$x_{t}=\mu +{\mathrm{Az}}_{t}+\varepsilon_{t},$$

where now ***A*** is a *p* × *q* matrix and the *p*‐variate noise *ε*_*t*_ is independent from the *q*‐variate signal ***z***_*t*_. Notice that in the econometric literature such model is known as dynamic factor model (see, e.g., Forni et al., 2000; Bai & Ng, 2002) which is, however, in contrast to the SOS approach usually considered in the case where *p* and *T* are increasing. An alternative noise model is the internal noise model (INM) given as

$$x_{t}=\mu +{\mathrm{Az}}_{t}=\mu +A_{s}z_{s,t}+A_{n}z_{n,t},$$

which basically says that the *p*‐variate latent component $z_{t}={\left(z_{s,t}^{\prime }z_{n,t}^{\prime }\right)}^{\prime }$ consists of a *q*‐variate signal ***z***_*s*,*t*_ which is independent form the (*p* − *q*) ‐variate noise **z**_*n*,*t*_. Here the mixing matrix has the corresponding partition into ***A*** = (***A***_*s*_ ***A***_*n*_).

In both models *q* is unknown and the goal is to estimate *q* together with the latent signals, for which in the context of this article the classical SOBI assumptions from Section 2 are made. In the ENM the noise is usually considered to be Gaussian white noise, that is, ***ε***_*t*_ ∼ *N*(**0**, *σ* ^2^ ***I***_*p*_) while in the INM also alternatives like white noise with a (*p* − *q*) ‐variate spherical distribution or *p* − *q* independent components without second‐order serial dependence were considered.

The main difference between INM and ENM is that consistent unmixing matrices for the signals can always be found, but in the ENM the recovered signals will always be contaminated by noise, which is not a problem in the INM.

#### 3.8.1 Estimation of *q* in ENM

The main idea to obtain *q* in the ENM is based on the eigenvector‐eigenvalue decomposition of Cov (***x***_*t*_) = ***UDU*** ^′^, where ***U*** is an orthogonal matrix and ***D*** is a diagonal matrix with eigenvalues *d*_1_, …, *d*_*p*_ ordered in decreasing order. Then, under ENM, these eigenvalues should be *d*_1_ ≥ ⋯ ≥ *d*_*q*_ > *σ* ^2^ = ⋯ = *σ* ^2^, that is, the *p* − *q* smallest eigenvalues should be equal to the noise variance. The decision about the number of informative components (*q*) is so far done mainly graphically using for example a scree plot. The estimate for the noise variance is then the average of the *p* − *q* smallest eigenvalues. Inference relating the eigenvalues makes either iid assumptions on the signal or requires the signals to be Gaussian in which case information theoretic criteria are available. For details see for example Wax and Kailath (1985); Zhao et al. (1986); Virta and Nordhausen (2019). In the dynamic factor model framework inference on *q* is studied in settings where both *p* and *T* are growing, that is, *p* → ∞ and *T* → ∞ (see, e.g., Forni et al., 2000; Bai & Ng, 2002; Onatski, 2009).

#### 3.8.2 Estimation of *q* in INM

In the INM all components which do not have second‐order serial dependence are considered as noise. For the purpose of this review we assume the most common case, that is, the noise is Gaussian white noise. Therefore, the main approach is to consider autocovariance matrices which should then have *q* nonzero eigenvalues. Usually the decision is then approached using successive tests for the null hypothesis

$$H_{0k}:\;z_{t}\;\text{contains}\;a\;\left(p-k\right)-\text{variate}\;\text{subvector}\;\text{of}\;\text{white}\;\text{noise}.$$

Again, as it is risky to base the decision here on one lag *τ* only, that is, on AMUSE alone, pooling information is again a safer choice. The pooled “SOBI” eigenvalues are defined as

$$\sum_{\tau \in T}\mathrm{diag}\left(\hat{U}{\hat{\mathrm{Cov}}}_{\tau }^{S}\left(x_{t}^{\mathrm{st}}\right){\hat{U}}^{\prime }\right)$$

and are ordered in decreasing order according to their squared values as the closest one to zero form the noise. Thus $\hat{U}$ has the partitioning ${\left({\hat{U}}_{s}^{\prime }{\hat{U}}_{n}^{\prime }\right)}^{\prime }$ identifying the signal and noise spaces.

The test statistic suggested in Matilainen et al. (2018); Virta and Nordhausen (2021) for this purpose is

$$t_{k}=\frac{1}{|T|{\left(p-k\right)}^{2}}\sum_{\tau \in T}{\left\|D_{\tau ,k}\right\|}^{2},$$

where $D_{\tau ,k}={\hat{U}}_{n}{\hat{\mathrm{Cov}\;}}_{\tau }^{S}\left(x_{t}^{\mathrm{st}}\right){\hat{U}}_{n}^{\prime }$. Matilainen et al. (2018) suggested bootstrapping strategies to get the distribution of *t*_*k*_ under *H*_0*k*_, and Virta and Nordhausen (2021) showed that under quite broad assumptions for a time series of length *T*, $T|T|{\left(p-q\right)}^{2}t_{q}$ converges to a $\chi_{|T|\left(p-q\right)\left(p-q-1\right)/2}^{2}$ distribution under *H*_0*q*_. Virta and Nordhausen (2021) also showed that successive testing can be used to get a consistent estimate for *q*.

Using stationary bootstrapping strategies for the pseudoeigenvalues of SOBI Nordhausen and Virta (2018) provided a ladle estimator for *q*. However, Virta and Nordhausen (2021) argued that successive application of the asymptotic test with a moderate set of lags is the preferable way to estimate *q*.

### 3.9 More sources than observed signals

In our second‐order separation model (1), the mixing matrix ***A*** is assumed to be a *p* × *p* square matrix, which implies the equality of the number of sources and observations. However, as described by Belouchrani et al. (1997), the SOBI method can still be applied if the mixing matrix ***A*** is a *p* × *q* matrix with *p* > *q*, that is, the dimension of sources is lower than the dimension of observations. If *p* < *q*, the SOBI method is not applicable since the diagonality of a nonsquare matrix is not well‐defined and thus joint diagonalization is no longer applicable. Lathauwer and Castaing (2008) has extended SOBI to a case where the number of unobserved signals is larger than the number of observed signals. This method is called as second‐order blind identification of underdetermined mixtures (SOBIUM) and it proceeds as follows.

In SOBIUM autocovariance matrices are first calculated and stacked into a third‐order tensor ℂ ∈ ℝ^*p* × *q* × *k*^, where *k* is the number of lags selected. Then, SOBIUM performs parallel factor analysis (PARAFAC) (Harshman & Lundy, 1994) for ℂ, that is, it finds vectors ***a***_1_, …, ***a***_*q*_, ***d***_1_, …, ***d***_*q*_ that satisfy

(21) $$\mathbb{C}=\sum_{i=1}^{q}\;a_{i}\circ a_{i}^{\prime }\circ d_{i},$$

where **a**_*i*_ represents each column of mixing matrix ***A***, ***d***_*i*_ is the *i*th column of the stacked ***D***_*τ*_ as defined in (3), and ∘ stands for element‐wise product (Hardmard product). The PARAFAC is usually performed using iterative algorithm such as alternating least squares (ALS) algorithm that minimizes the cost function

(22) $$f\left(a_{i},d_{i}\right)=∥\mathbb{C}-\sum_{i=1}^{q}\;a_{i}\circ a_{i}^{\prime }\circ d_{i}{∥}^{2}$$

(Kroonenberg & De Leeuw, 1980). Enhanced line search (ELS) proposed by Rajih et al. (2008) provides improved computational efficiency when compared to PARAFAC.

The SOBIUM algorithm is shown to be identifiable when *p* ≤ *q* ≤ 2*p* − 2 and the number of selected lags *k* ≥ *q*. There also exists an enhanced version of SOBIUM that is specifically designed for *k* ≥ *q* (Lathauwer & Castaing, 2008).

## 4 AN APPLICATION OF SOBI AND RELATED METHODS

**FIGURE 3:**
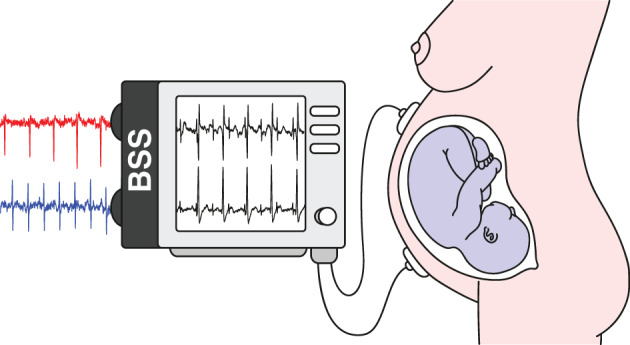
Schematic representation of an electrocardiography recording with two sensors for a pregnant women. BSS, blind source separation

**FIGURE 4:**
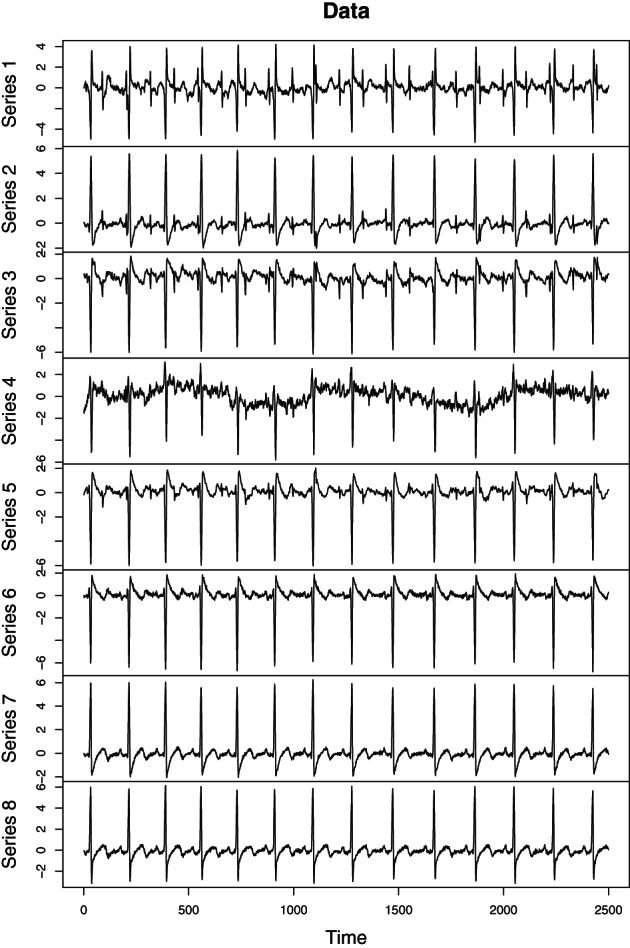
Electrocardiography recordings of a pregnant women using eight sensors

**FIGURE 5:**
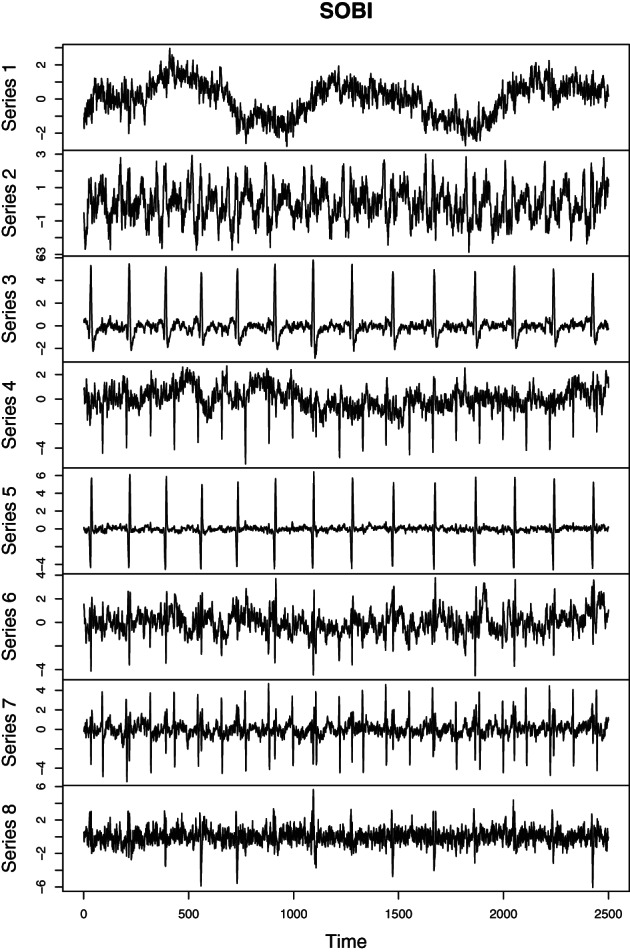
Electrocardiography latent source components using second‐order blind identification (SOBI) with $T=\left\{1,\ldots ,12\right\}$

**FIGURE 6:**
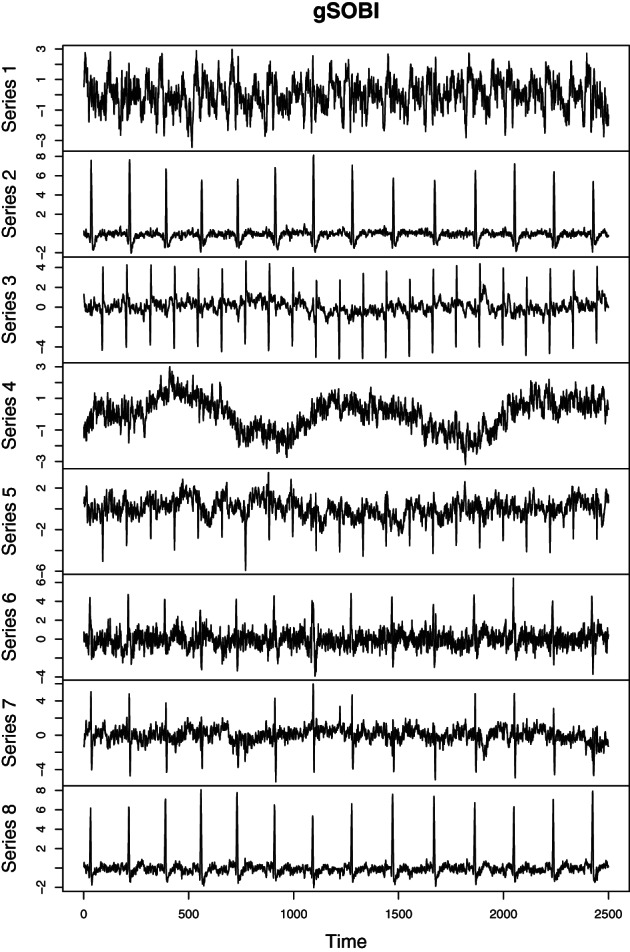
Electrocardiography latent source components using generalized second‐order blind identification (gSOBI) with *w* = 0.9, $T_{1}=\left\{1,\ldots ,12\right\}$, and $T_{2}=\left\{1,2,3\right\}$

As mentioned earlier SOBI and related methods are used in many applied fields. In the following we consider electrocardiography (ECG) recordings of a pregnant women. The goal of the analysis is to separate the heartbeats of the fetus. In an ECG recording sensors are placed on the skin of the mother measuring the electrical potential generated by the heart muscles. Figure 3 gives a schematic representation of a recording with two sensors. Here the two sources illustrate heartbeats of the mother and her fetus. The fetal heart rate is expected to be higher than that of the mother. The two sensors measure mixtures of the two heartbeats, and the weights can be seen as a measure of proximity to the corresponding heart. The goal of SOS is to separate the heartbeats of the fetus from those of the mother's based on the observed mixtures. As also other muscles in the body create electrical potential, which can be considered as artifacts, more than two sensors will in practice be used in actual ECG recordings. De Lathauwer et al. (1995) consider a recording that uses eight sensors, five in the stomach area and three in the chest area. The data are available for example in the supplement of Miettinen et al. (2017). The observed ECG measurements are given in Figure 4. Figure 5 depicts latent sources based on SOBI with $T=\left\{1,\ldots ,12\right\}$. As seen in the figure, one clear heartbeat pattern is seen, but not two. Thus, we explore the results based on gSOBI using *w* = 0.9, $T_{1}=\left\{1,\ldots ,12\right\}$ and $T_{2}=\left\{1,2,3\right\}$. Estimated sources based on gSOBI are visualized in Figure 6. Series 3 seems to correspond to the heartbeat of the fetus being twice as fast as the mother's heartbeat in Series 2. This example illustrates the case where also the higher moments include crucial information on the time series.

## 5 ALGORITHMS AND IMPLEMENTATIONS OF SOBI AND RELATED METHODS

The estimation of SOBI unmixing matrix is simple when data are moderate in size. However, as typical applications, such as those encountered in neurosciences, usually generate huge datasets, computational issues have got more attention in recent years. The impact of the used (approximate) joint diagonalization algorithm on the estimation has been considered in Illner et al. (2015); Kalogiannis et al. (2017). Possibilities for an online variant of the SOBI algorithm, where the observations arrive successively, have been considered in Zhang et al. (2015). Parallel implementations of SOBI are studied in Li et al. (2019).

As the development of SOS methods is driven by basically two communities, signal processing and statistics implementations of the corresponding methods are usually available in the preferred software of these communities, that is, MATLAB (MATLAB, 2019) and R (R Core Team, 2020), respectively.

For Matlab, a nonexhaustive list of implementations of some of the above methods can be found from the EEGLAB toolbox (Delorme & Makeig, 2004), ICALAB toolbox (Cichocki et al., 2007) or BSSGUI (Petkov & Koldovský, 2009), for example. In R, especially more current methods are available in the packages JADE Miettinen et al. (2017), BSSasymp (Miettinen et al., 2017), tsBSS (Matilainen et al., 2019) and tensorBSS (Virta et al., 2020b).

A machine learning library running on many platforms and containing for example SOBI is SHOGUN (Sonnenburg et al., 2017).

## 6 CONCLUSIONS

In this article we reviewed the latest developments in the area of SOS. SOS methods originate from signal processing literature, and, as seen in this article, the classical SOBI method has been extended since its appearance to various different time series settings. Besides signal processing community, the SOS methods have recently gained interest also among statisticians. The statistical properties of two well established SOBI unmixing matrix estimators, the deflation‐based estimator and the symmetric estimator, are derived in Miettinen et al. (2014, 2016). Such derivations are useful when one, for example, wishes to develop a method that aims at more efficient signal separation result. We present in this article two efficient methods (eSOBI and aSOBI), which utilize asymptotic results of SOBI estimator. Some theoretical results for the generalized SOBI estimator (gSOBI) can also be found in literature (Miettinen, Matilainen, et al., 2020; Miettinen, Vorobyov, & Ollila, 2020). However, the statistical properties of other variants of SOBI remain mostly unknown and need to be studied.

As modern datasets are often high‐dimensional and highly complex, more sophisticated SOBI methods for such data need to be developed. We already discussed here the methods developed recently for matrix‐valued and tensor‐valued data (see, e.g., Virta & Nordhausen, 2017b, and references therein). Other directions for future research include developing methods that can handle spatial and spatiotemporal dependencies as well as methods that are suitable for functional data. Notice that in Bachoc et al. (2020) the first step toward modeling spatial data was taken. When high‐dimensional data are measured the main aim of the analysis is often to separate the signals of interest from noise components. Recent developments in this area can be found in Virta and Nordhausen (2021).

This review focused on the case where the mixing is instantaneous. However, there exists also many scenarios where it would be natural to consider that at time point *t* the observed signal is a mixture of current and past latent values. For example, in sound applications there might be in addition to the current sounds also some echos present. In the signal processing community such models are known as convolutive blind source separation models and are reviewed in Castella et al. (2010), for example. In this context, one can also mention the dynamic factor model (DFM) considered in Forni et al. (2000); Bai and Ng (2002); Stock and Watson (2002b, 2002a); Forni et al. (2004); Forni et al. (2017), among others. DFMs can also be considered in a convolution framework and can be combined with the external noise model mentioned earlier and generalized thereof. DFMs, with the special case dynamic principal component analysis (Brillinger, 1974), work usually in the frequency domain and can be applied also when the dimension is very high, unlike the SOS models discussed in this article, which usually assume the number of components to be much smaller than the number of available time points. The connection between the models and methods proposed in the DFM literature with those proposed in signal processing community need definitely careful consideration in future research. Finally, one issue that seems to be understudied in the field of SOS, is robustness. Robustness is a major issue for multivariate analysis because outliers become increasingly common as the dimension of data increases. However, as seen in this article, only few robust proposals for SOS exist in the literature.

## AUTHOR CONTRIBUTIONS

**Yan Pan:** Methodology. **Markus Matilainen:** Methodology. **Sara Taskinen:** Methodology. **Klaus Nordhausen:** Methodology.

## CONFLICT OF INTEREST

The authors have declared no conflicts of interest for this article.

## RELATED WIREs ARTICLES

Independent component analysis: A statistical perspective

Tensor decompositions and data fusion in epileptic electroencephalography and functional magnetic resonance imaging data
